# Case report: *Mycobacterium neoaurum* infection during ICI therapy in a hepatocellular carcinoma patient with psoriasis

**DOI:** 10.3389/fimmu.2022.972302

**Published:** 2022-08-22

**Authors:** Ling Pang, Zhongju Chen, Dong Xu, Weiting Cheng

**Affiliations:** ^1^ Department of Infectious Disease, Tongji Hospital, Tongji Medical College, Huazhong University of Science and Technology, Wuhan, China; ^2^ Department of Laboratory Medicine, Tongji Hospital, Tongji Medical College, Huazhong University of Science and Technology, Wuhan, China; ^3^ Department of Oncology, Wuhan No1. Hospital, Wuhan, China

**Keywords:** immune checkpoint inhibitor (ICI), *mycobacterium neoaurum* infection, psoriasis, hepatocellular carcinoma, case report

## Abstract

We report here a patient with advanced hepatocellular carcinoma (HCC) and psoriasis treated with immune checkpoint inhibitor (ICI) therapy who experienced tumor partial response and psoriatic exacerbation. Meanwhile, the patient contracted *mycobacterium neoaurum* during the treatment period, while it was an opportunistic infection and mainly happened in immunosuppressed patients. We discussed the possibility that this infection was an ICI-associated infection independent of immunosuppression due to dysregulated immunity, which was the result of the effects of immunotherapy and autoimmune disease (AID), and the characteristics and treatment of *M. neoaurum*, which was rarely reported in China. This case highlights the fact that some infections can be precipitated by ICIs in the absence of immunosuppressive treatment, especially the patients with AID.

## Introduction

Immune checkpoint inhibitors (ICIs), including anti-cytotoxic T lymphocyte antigen 4 (CTLA-4), anti-programmed cell death 1 (PD-1), and anti-programmed cell death 1 ligand 1 (PD-L1) antibodies, are novel agents approved for the treatment of late-stage malignancies in recent years, including advanced hepatocellular carcinoma (HCC). Sorafenib has been the only systemic treatment option for patients with advanced HCC in the past (1). At present, combination treatment with an ICI and an anti-vascular endothelial growth factor (anti-VEGF) agent has been an effective strategy (2). However, the majority of patients suffer from immune-related adverse events (irAEs), with an incidence between 54% and 76%, according to a metanalysis of trial data (3), especially in patients with autoimmune disease (AID) (4), so irAEs partly restrict their use. Moreover, the precise mechanism underlying irAEs is unknown, maybe due to dysregulated immunity (5).

Here, we report the first case of *Mycobacterium neoaurum* infection in China mainland, in an adult man with advanced HCC and psoriasis under ICI immunotherapy. We believe that dysregulated immunity caused by immunotherapy and AID counterintuitively favors the pathogen.

## Patient description

A 53-year-old Chinese man has more than 20 years of history of chronic hepatitis B virus (HBV) infection and psoriasis without standardized examination and treatment in the past. Other than that, no other underlying diseases. In January 2021, he was admitted with psoriasis. At that time, he had minor psoriatic lesions on his trunk and extremities. Chest computed tomography (CT) happened to find multiple low-density lesions in the liver. Subsequently, the liver magnetic resonance imaging (MRI) showed multiple substantial lesions, which were suspected neoplastic lesions. It was identified as HCC by testing hepatic puncture biopsy. Hilar hepatic and retroperitoneal lymph nodes metastasis were observed in positron emission tomography/computed tomography (PET/CT). Alpha-fetoprotein was significantly elevated (>1200 μg/L). The patient was Child-Pugh A with elevated HBV DNA load with an Eastern Cooperative Oncology Group (ECOG) performance status of 0. And immunohistochemistry of tumor tissue showed AFP (±), CD34 (vascular +), CK19 (–), CK20 (–), CK7 (scattered +), CK-P (+), GPC (-), Hepatocyte (+), Ki-67 (LI about 10%), Vimentin (-). CNLC IIIb HCC was diagnosed in February 2021.

He was started on anti-hepatitis B virus therapy. Treatment for HCC did not begin with lenvatinib until his HBV DNA load dropped to normal levels. Two months later, the treatment regimen was adjusted to lenvatinib in combination with camrelizumab. The assessment of efficacy is partial remission (PR) after completing the combination for three cycles.

**Figure 1:**
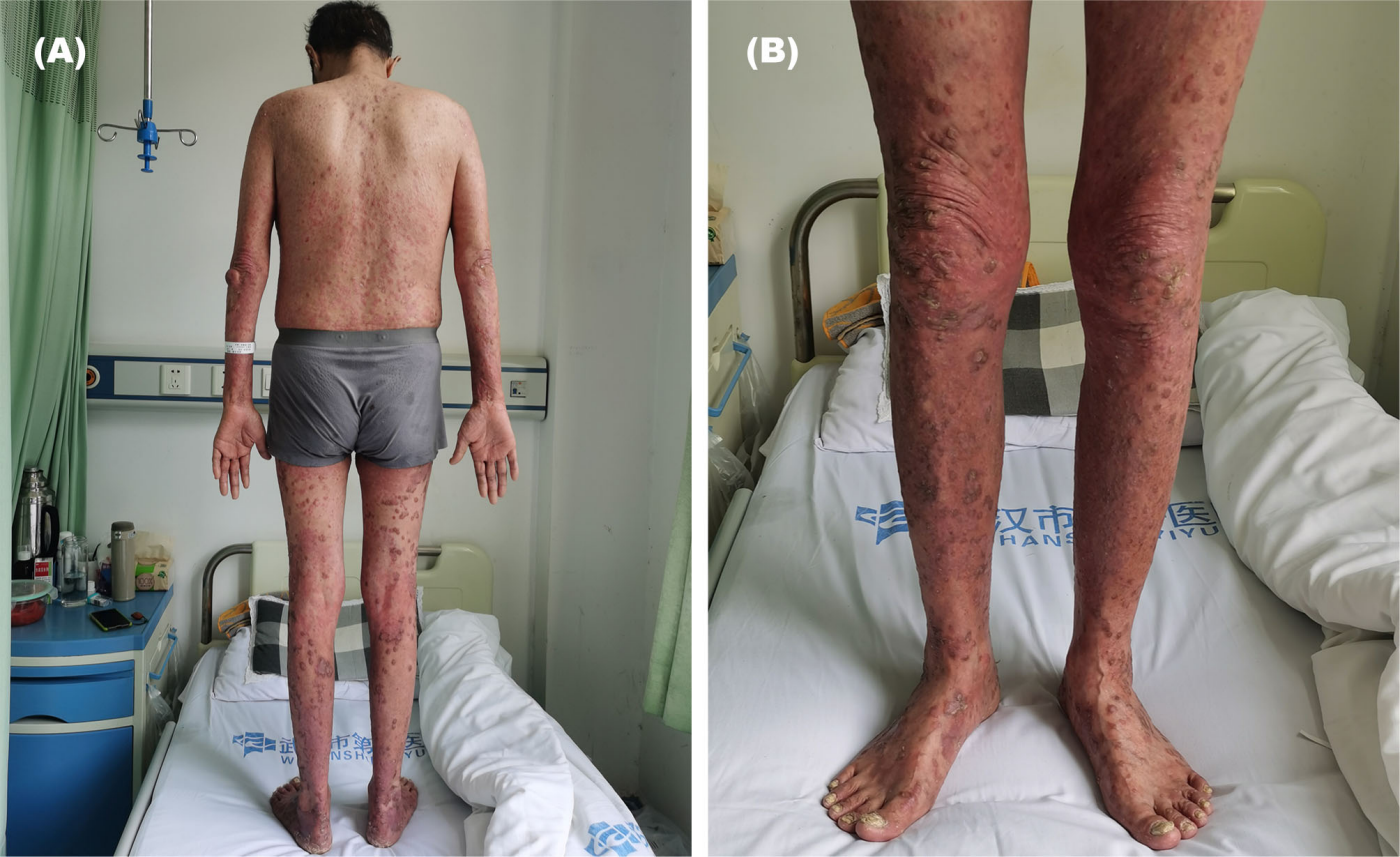
Psoriasis exacerbated during the 6th course of anti-programmed cell death 1 (PD-1) (camrelizumab), which characterized by skin lesions expanding on both hands, legs, and trunk. (**A**, back of the whole body), (**B**, front of the legs).

**Figure 2:**
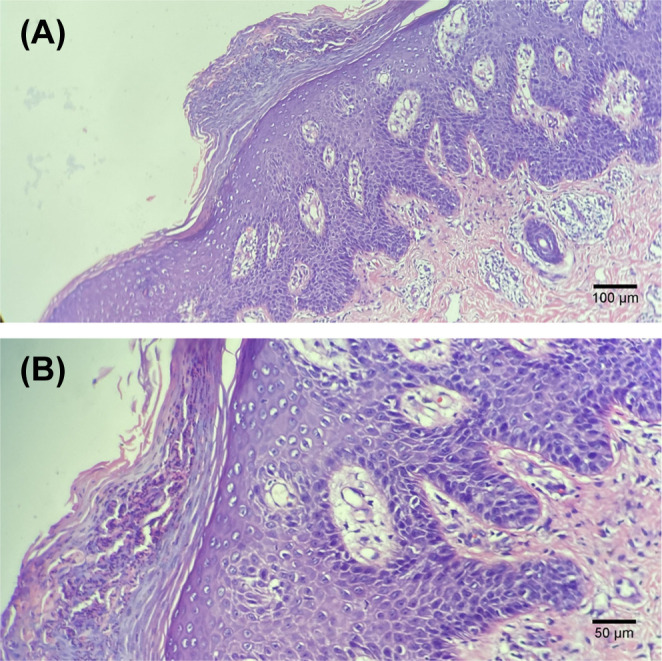
Histological examination of the skin biopsy showing typical features of psoriasis, i.e. thickened epidermis with hyperkeratosis and regular acanthosis, neutrophils trapped in the stratum corneum (Munro micro-abscesses), considerable oedema of the dermis and slight lymphocyte infiltrate around dermal capillaries. Hematoxylin and eosin staining; (**A**, 100× images), (**B**, 200×images).

During the 6th course of camrelizumab, MRI suggested liver tumor progression. At that time, his psoriasis progressed with obvious pain and skin lesions expanding on both hands, legs, and trunk (**Figure 1**). We treated with topical corticosteroids and body balm and withdraw the next immunotherapy. Subsequently, a minor clinical improvement of the psoriatic lesions was noted. Finally, a skin biopsy confirmed the diagnosis of psoriasis (**Figure 2**).

**Figure 3:**
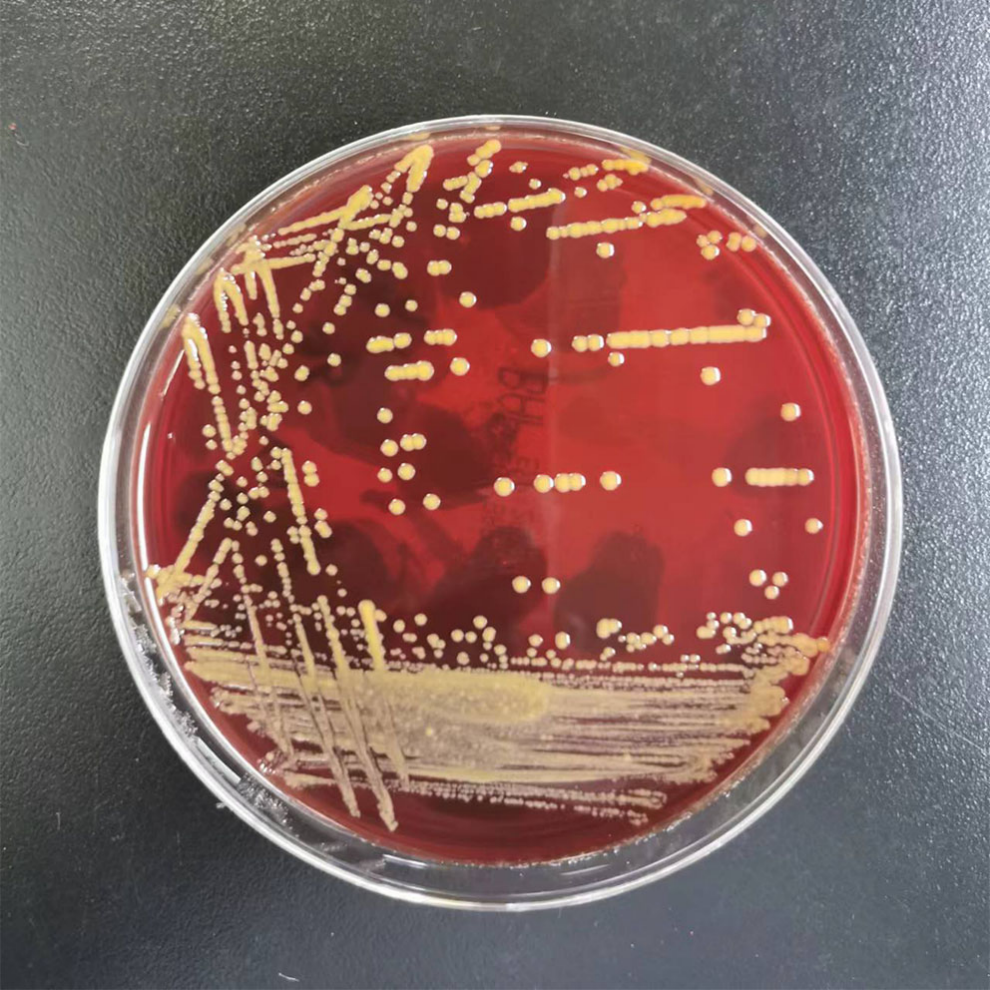
Mycobacterium neoaurum growth on blood plate. Note the smooth daffodil yellow appearance of colonies.

After the 6th course of camrelizumab, the patient began to experience intermittent fatigue and chills without fever and blood tests were normal. He presented to the hospital for the 8th course on schedule. His laboratory results showed normal WBC count and differential at the time of admission, except for slightly high monocytes. But suddenly, he had a fever of 39°C and a loss of appetite. We did a blood culture, and his symptom gradually improved without any treatment. Seven days later, the blood culture grew *M. neoaurum* (**Figure 3**). Given the limitations like diagnostic qualifications and laboratory biosafety, the patient went to another two hospitals, and the same results were reported in multiple blood cultures. Antibiotic susceptibility testing was performed on this isolate, suggesting that it was resistant to all commonly used anti-mycobacterial drugs including ceftriaxone, imipenem, amikacin, ciprofloxacin, levofloxacin, sulfamethoxazole-trimethoprim, and ertapenem. Considering his skin damage and contact with aquatic products, we thought that *M. neoaurum* bacteremia may be from a cutaneous infection. The optimal therapy for infections caused by *M. neoaurum* has not been established, and the single drug is also exploratory, providing individualized treatment experience for future treatment and reducing drug resistance. Furthermore, the patient strongly requested to keep the infusion port when we informed the patient that may be from the implantable venous access port (PORT). Thus, the patient received oral contezolid and suspended camrelizumab during treatment for *M. neoaurum* infection. Ten days later, the blood culture was negative. Two cycles of contezolid were continued due to bacteremia.

Unfortunately, his HCC significantly progressed 2 months after stopping immunotherapy, and distant osseous metastases were found. Thus, a single fraction of palliative radiotherapy was delivered to bone lesions (40 Gy) to alleviate his pain. Systemic therapy with camrelizumab continued. Four weeks later, he fevered again, and two sets of blood culture specimens, from the PORT, implanted for 11 months, and a peripheral vein respectively, were collected. The blood culture collected from the POPT grew *M. neoaurum*, and his catheter was reluctantly and immediately removed. He received contezolid for 2 weeks again with the resolution of bacteremia.

## Discussion

To our knowledge, this is the first case of *M. neoaurum* bacteremia during ICI therapy. ICIs theoretically do not seem to directly increase the risk of infection (6) but rather reactivate the cytotoxic T cells that were suppressed by the cancer cells to attack the tumor cells and enhance the immune system (7, 8). It was believed that concomitant use of immunosuppressants including systemic corticosteroids with ICIs treatment increased infection risk (9). However, research has found that no significant difference in the use of immunosuppressive agents between patients with and without infections (10, 11). Morelli et al. suggested that the role of a dysregulated immunity whose mounted response paradoxically favors the pathogen (9). We think that it may be immunotherapy infection due to dysregulated immunity, especially when the patient with psoriasis, which can exacerbate immune disorders.

*M. neoaurum*, a member of nontuberculous mycobacteria (NTM), found in the environment, particularly in water and soil, was initially isolated from soil by Tsukamura in 1972 (12) and then described as a human pathogen for the first time in 1988 (13). So far there are only 28 cases from the literature to define the management of this rare infection. *M. neoaurum* infection mainly causes catheter-associated bloodstream infection (CABI) and develops in immune-compromised populations (14, 15). Our patient was diagnosed with CABI without immunocompromised, but we believed that ICIs seem to directly increase the risk of infection and the hyperinflammatory dysregulated immunity associated with ICIs drives this infection. Furthermore, in this case, the drug sensitivity test suggested all commonly used anti-mycobacterial drugs resistance, and we thought it was CABI after ruling out the source of skin infection, we removed the catheter and applied contezolid, a new generation of oxazolidinone antibiotics developed independently in China, with antibacterial activity similar to linezolid (16). In contrast, contezolid has fewer adverse effects on myelosuppression and better clinical application. Considering that the optimal therapy for infections caused by *M. neoaurum* has not been established and the single drug is also exploratory, we try to provide an individualized treatment experience for future treatment and reduce drug resistance in this case.

Before being diagnosed with advanced HCC, the patient had psoriasis for many years, without systemic therapy. However, lesions exacerbated during the 6th course of camrelizumab. The safety and efficacy of ICIs in patients with cancer and pre-existing AID are still a key issues to be addressed. Several studies have suggested that patients with AID may easier to occur exacerbation of AID and irAEs (4, 14, 15, 17), while in 56 patients with non-small cell lung cancer (NSCLC) with AID treated with a PD-(L)1 inhibitor, the incidence of irAEs was similar to reported rates in clinical trials where patients with AID were excluded (12). In addition, based on the current evidence from retrospective studies, most irAEs were manageable with corticosteroids in these patients (4). Thus, there seems no reason to exclude these patients from cancer immunotherapy even though patients with AID may be at higher risk of developing irAEs or have more severe irAEs. A systematic review suggested that half of the patients with AID had exacerbation of pre-existing AID, generally had the same manifestations as those occurring before ICI therapy, and the most commonly reported AID was psoriatic arthritis and/or psoriasis (22.8%) (4). Close monitoring and timely treatment can deal with most problems (13). Certainly, future prospective studies are needed to provide more favorable evidence.

Our patient, with advanced HCC and psoriasis under ICI immunotherapy, suffered *M. neoaurum* infection. Although there was a lack of more indicators to assess the immune status of the body, we think that the hyperinflammatory dysregulated immunity associated with ICI drove this special pathogen infection, which supported the hypothesis that immune dysregulation under ICI predisposes patients to opportunistic infections including *M. neoaurum*. Clinical vigilance and early diagnosis are important to prevent severe infection and continuity of anti-tumor treatment. On the other hand, there is still a debate about whether patients with AID can receive ICIs treatment. IrAEs in patients with AID, who are receiving ICIs, can often be managed without discontinuing therapy, although some events may be severe and fatal (4, 18, 19). Moreover, close monitoring irAEs, including the occurrence of irAEs and the flares of the AID, and a multidisciplinary approach should be realized.

## Patient perspective

During the whole process, the patient and his wife were informed about treatment options, risk, and possibility of relapse. They were aware of the complexity of his unusual case, and the patient provided written informed consent for the publication of his case.

## Data availability statement

The original contributions presented in the study are included in the article/supplementary materials. Further inquiries can be directed to the corresponding author.

## Ethics statement

Written informed consent was obtained from the individual(s) for the publication of any potentially identifiable images or data included in this article.

## Author contributions

All authors listed have made a substantial, direct, and intellectual contribution to the work, and approved it for publication.

## Conflict of interest

The authors declare that the research was conducted in the absence of any commercial or financial relationships that could be construed as a potential conflict of interest.

## Publisher’s note

All claims expressed in this article are solely those of the authors and do not necessarily represent those of their affiliated organizations, or those of the publisher, the editors and the reviewers. Any product that may be evaluated in this article, or claim that may be made by its manufacturer, is not guaranteed or endorsed by the publisher.
